# Cross-sectional study on the association of periodontitis with arterial hypertension in the Hamburg City Health Study

**DOI:** 10.1186/s40001-022-00811-y

**Published:** 2022-09-16

**Authors:** Henrieke Könnecke, Renate B. Schnabel, Carolin Walther, Ragna Lamprecht, Guido Heydecke, Udo Seedorf, Annika Jagodzinski, Katrin Borof, Tanja Zeller, Thomas Beikler, Ralf Smeets, Martin Gosau, Christian-Alexander Behrendt, Ulrich Wenzel, Christin S. Börschel, Mahir Karakas, Stefan Blankenberg, Ghazal Aarabi

**Affiliations:** 1 Department of Prosthetic Dentistry, Center for Dental and Oral Medicine, University Medical Center Hamburg-Eppendorf, Martinistrasse 52, 20251 Hamburg, Germany; 2 Department of Cardiology, University Heart and Vascular Center, Hamburg, Germany; 3 DZHK (German Center for Cardiovascular Research), Partner Site Hamburg, Kiel, Lübeck Germany; 4 Epidemiological Study Center, University Medical Center Hamburg-Eppendorf, Hamburg, Germany; 5 Department of Periodontics, Preventive and Restorative Dentistry, University Medical Center Hamburg-Eppendorf, Hamburg, Germany; 6 Department of Oral and Maxillofacial Surgery, University Medical Center Hamburg-Eppendorf, Hamburg, Germany; 7 Department of Oral and Maxillofacial Surgery, Division of „Regenerative Orofacial Medicine”, University Medical Center Hamburg-Eppendorf, Hamburg, Germany; 8 Department of Vascular Medicine, University Medical Center Hamburg-Eppendorf, Hamburg, Germany; 9 III. Department of Medicine, University Medical Center Hamburg-Eppendorf, Hamburg, Germany

**Keywords:** Periodontitis, Hypertension, Blood pressure, Epidemiology, Cross-sectional studies

## Abstract

**Aim:**

Aim of this study was to investigate the association between periodontitis and arterial hypertension, both of which show correlations with classical cardiovascular risk factors and inflammatory activity.

**Materials and methods:**

A cross-sectional analysis of data from a large population-based health survey (the Hamburg City Health Study, HCHS) including 5934 participants with complete periodontal examination and blood pressure data, of whom 5735 had medical records regarding anti-hypertensive medication, was performed. Probing depths, gingival recessions, bleeding on probing (BOP), dental plaque, and decayed-missing-filled teeth (DMFT) indices were recorded as measures of oral health. Clinical attachment loss (CAL) per tooth was calculated and periodontitis was staged into three groups (no/mild, moderate, severe). Arterial hypertension was diagnosed based on the participants’ medication history and systolic and diastolic blood pressure values. Logistic regression models were constructed accounting for a set of potential confounders (age, sex, smoking, body mass index (BMI), diabetes, educational level, alcohol intake) and high sensitivity-C-reactive protein (hsCRP).

**Results:**

The odds of arterial hypertension increased significantly along with periodontitis severity (OR for severe periodontitis: 2.19; 95% CI 1.85–2.59; *p* < 0.001; OR for moderate periodontitis: 1.65; 95% CI 1.45–1.87; *p* < 0.001). Participants with moderate or severe periodontitis also had significantly higher age- and sex-adjusted odds of arterial hypertension, which was slightly weakened when additionally adjusted for BMI, diabetes, smoking, educational level, and alcohol intake (OR for severe PD: 1.28, 95% CI 1.04–1.59, *p* = 0.02; OR for moderate PD: 1.30, 95% CI 1.11–1.52, *p* = 0.001). The fraction of participants with undertreated hypertension (untreated and poorly controlled hypertension) was considerably larger in participants with severe periodontitis than in those with no/mild periodontitis (50.1% vs. 37.4% for no/mild periodontitis).

**Conclusions:**

The study shows an association between periodontitis and arterial hypertension that is independent of age, sex, diabetes, BMI, smoking, educational level, and alcohol intake. In addition, undertreatment of hypertension was more common in people with severe periodontitis compared with periodontally more healthy people.

**Supplementary Information:**

The online version contains supplementary material available at 10.1186/s40001-022-00811-y.

## Introduction

Arterial hypertension refers to a condition of consistently elevated blood pressure, which is specified as systolic blood pressure (SBP) of 140 mmHg or higher, or diastolic blood pressure (DBP) of 90 mmHg or higher [1, 2]. It was estimated that the global age-standardized prevalence of arterial hypertension was 24% in men and 20% in women in 2015 [3]. Established risk factors for the development of arterial hypertension are male sex, increased age, obesity, physical inactivity, stress, high salt intake, high alcohol consumption and predisposing genetic factors [4, 5]. There are two traditional working hypotheses for the mechanisms leading to arterial hypertension. The first is the “kidney-centric” hypothesis proposed by Guyton [6], which states that the kidney is crucial in mediating the relationship between salt and hypertension. The second hypothesis stresses the critical importance of the control of peripheral vascular resistance [7]. Increasing evidence indicates that hypertension and hypertensive end-organ damage are not only mediated by hemodynamic injury but also by inflammation [8–12], such as periodontitis (PD) [13], which in its severe form has a prevalence of about 14% among the global adult population [14]. PD is a bacterially induced chronic inflammation that leads to an irreversible destruction of the periodontium caused by a dysbiotic microflora, which can trigger an overaggressive host response [15]. Previous studies have identified *Porphyromonas gingivalis*, *Aggregatibacter actinomycetemcomitans* and *Tannerella forsythia* as causative agents of PD [16–18]. It could recently been demonstrated that the oral microbiome’s ability to reduce inorganic nitrate to nitrite and nitric oxide may provide a plausible mechanism linking PD to arterial hypertension [19].

A number of previous epidemiological studies and meta-analyses support that patients with PD have higher mean blood pressure compared with people without PD and that severe PD is associated with hypertension [20–24]. However, the effect sizes observed in individual studies varied widely in relation to the severity of PD and the dependence on sex, the influence of anti-hypertensive medication, and the role of confounding were not well-established according to a recently published meta-analysis [24]. Thus, we were interested in examining the association between PD and blood pressure in a large population-based sample of middle-aged and older men and women recruited between February 2016 and November 2018 in Hamburg, Germany, with special emphasis on PD severity staging, potential confounders, and antihypertensive medication. Since evidence that is based on prospective analyses is still scarce, this cross-sectional analysis should also serve as a starting point for future prospective analyses.

## Methods

### Subjects, study design and setting

The Hamburg City Health Study (HCHS) is a large, population-based cohort study conducted by the University Medical Center Hamburg–Eppendorf (UKE). Major chronic diseases, such as myocardial infarction, atrial fibrillation, stroke, or dementia are studied to understand their risk factors by relating the effects of genetics, previous diseases, lifestyle and environmental conditions on health and disease [25].

The HCHS started in spring 2016 and is projected to include a random sample of 45,000 inhabitants aged from 45 to 74 years over a period of 6 years. General inclusion criteria are an adequate knowledge of the German language as well as physical and psychological capability to take part in the investigation. This study represents a cross-sectional evaluation of the baseline data from the first 10,000 participants.

The recruitment procedure was described in Ref. [25]. Briefly, potential participants aged 45–74 years were identified from a random sample from the official inhabitant data file (*N* = 45,000) divided into six age brackets stratified by men and women. The participants received an invitation letter to their home address and an information leaflet providing basic study information. Participants were asked to organize their own appointment at the epidemiological study center at the University Medical Center Hamburg–Eppendorf. The appointment was initiated by a study nurse explaining the study rationale and participants were asked to sign informed consent forms including study participation, an extraction of a skin punch to create induced pluripotent stem cells and either none, one or all of the following options: external, virtual or internal autopsy in the event of death. In the end, participants also signed a consent accepting that both double de-identified and pseudo-anonymized data may be transferred to cooperation partners. Participants were also asked for consent to match their health insurance and pension insurance data with the HCHS data set. During a 7-h examination participants underwent validated examinations of different organ systems. The participation rate was 65%. The dental sub-cohort consisted of 6209 participants. Blood pressure values were available for 5934 participants, which were included (for details see Additional files 1 and 2).

The article was written in accordance with recommendations issued by the “The Strengthening the Reporting of Observational Studies in Epidemiology (STROBE) Initiative” [26].

### Variables

#### Assessment of arterial hypertension

The diagnosis of arterial hypertension was based on a detailed medical history, with focus on antihypertensive drugs, a blood pressure measurement and patients' self-report. Blood pressure was measured by calibrated and certified study nurses on the right arm after 5 min rest for two times sitting. The average of two measurements of SBP was calculated. High blood pressure was defined according to the World Health Organization as SBP ≥ 140 mmHg and/or DBP ≥ 90 mmHg. Participants were classified in four groups: (1) ‘’healthy’’ participants with no sign for arterial hypertension (SBP ≤ 139 mmHg). (2) Hypertensive participants with a positive drug history and SBP values within the normal range were categorized as "controlled"; (3) ‘untreated hypertensive participants (SBP ≥ 140), and (4) participants with a positive drug history and high blood pressure (SBP ≥ 140) were categorized as ‘’poorly controlled’.

#### Oral examination

The periodontal examination, which took about 30 min, was performed by trained and calibrated examiners following a pre-specified SOP under the supervision of a dentist as described in Ref. [27]. Probing was performed with a PCP UNC 15 periodontal probe (Hu-Friedy, Chicago, IL, USA). Briefly, 49 examiners, who were also involved in conducting the oral health examinations for the German National Cohort (GNC) study, collected the raw data, such as number of teeth, pocket depths, number of bleeding points on probing etc., which were used by two dentists to establish the diagnosis. In case of disagreement, consensus was established by consulting a third dentist. Data accuracy was established by regular training and calibration of the staff in the pilot phase of the study and while the study was ongoing. Electronic data capture and transfer, longitudinal performance evaluation, and statistical monitoring were performed regularly during the study. The validity of the results obtained by trained non-dental examiners was confirmed in a published quality control study by Holtfreter et al. on the basis of data from the GNC study [28]. The plaque index (PI) was measured at two interproximal sites as a variant of the index described in Ref. [29] and the bleeding-on-probing index (BOP) was determined at one site per tooth as a variant of the index described in Ref. [30].

The probing depths of the periodontal pockets and the recessions were measured at six sites per tooth and the clinical attachment loss (CAL = probing depth + recession) was calculated for each tooth. The degree of PD severity was staged according to the criteria of Eke and Page [31] as described by Lamprecht et al. [27]. In addition, the decayed-missing-filled-teeth (DMFT) index was determined by trained and calibrated examiners. Training and calibration was performed by two experienced dentists by photograph and slide-based training following by in vivo training and calibration exercises. The in vivo training sessions allowed examiners to discuss and evaluate their scoring of the in vivo presentation of decayed, missing, and filled teeth before data collection. The calibration process started with an examination of a test person performed by an experienced dentist who set the “gold standard”. Subsequently, several examinations on the same person were performed by up to five different examiners. The scoring of each examiner was recorded and the calibration ended only successfully if the examiner was able to achieve 100% agreement with the “gold standard” in at least five different test persons.

#### Confounders

Each participant completed validated self-report questionnaires before (lifestyle, environment, family, sociodemographic and educational characteristics, age, nationality, sex,) and during (medical backgrounds, medical and family history of widespread chronic diseases including diabetes) the baseline visit [32]. Smoking behaviour was assessed using the Fagerström questionnaire [33]. The body mass index (BMI) expressed as kg/m^2^ (normal range: 18.5 bis 24.9) and waist-to-hip ratio (normal range: ≤ 1.0 for men and ≤ 0.85 for women) were calculated. In addition, high-sensitive C-reactive protein (hsCRP, normal range: < 0.5 mg/dL), fasting glucose (normal range: 70–99 mg/dL) and triglycerides (normal range: ≤ 150 mg/dL), HDL- (40–60 mg/dL), LDL- (< 116 mg/dL) and total cholesterol (< 200 mg/dL), were measured by routine laboratory tests.

### Statistical analysis

Basic characteristics were summarized for each status of PD with frequency distributions for the categorical variables and median (interquartile range [IQR]) for continuous variables). The Kruskal–Wallis test was applied to compare central tendency measures and differences in prevalence were assessed by the chi-square test. Crude and progressively adjusted logistic models were used to evaluate the association between the periodontal status (exposure) and arterial hypertension (outcome). Hypertension was modelled as dichotomic dependent variable defined by systolic blood pressure of 140 mmHg or more and/or diastolic blood pressure of 90 mmHg or more and/or current use of antihypertensives. Independent variables were selected among clinical and demographic characteristics and included age, sex, BMI, diabetes, smoking, education, and alcohol intake. Following the initial crude model (model 1, unadjusted), three extended models were generated with adjustment for age and sex (model 2), additional inclusion of hsCRP after log-transformation (model 3), and an additional model including age, sex, BMI, diabetes, smoking, educational level, and alcohol intake (model 4). The results of each model were expressed as odds ratio per standard deviation (OR per SD) and a *p* value < 0.05 was considered statistically significant. All statistical analyses were performed using R software, version 4.0.5 (R Project for Statistical Computing).

## Results

### Characteristics of participants

Table 1 presents distributions of baseline characteristics of the study participants stratified by PD severity grades (none/mild *N* = 1453 [women *N* = 878]); (moderate *N* = 3580 [women *N* = 1814]); (severe *N* = 1176 [women *N* = 460]).

**Table 1:** Characteristics of the study population

Periodontitis	None/mild	Moderate	Severe	*p* value
	1453 (23.4)	3580 (57.7)	1176 (18.9)	
	Median [IQR] or *n* (%)			
*Socio-demographic characteristics*				
Sex = female	878 (60.5)	1814 (50.7)	460 (39.1)	< 0.001
Age (years)	59 [52, 66]	63 [55, 69]	66 [59, 71]	< 0.001
Education				< 0.001
Low	43 (3.2)	151 (4.4)	55 (5)	
Medium	675 (48.4)	1711 (50.0)	605 (54.5)	
High	675 (48.4)	1559 (45.6)	450 (40.5)	
*Risk factors*				
BMI (kg/m^2^)	25.56 [23.03, 28.67]	26.02 [23.55, 29]	26.45 [24.14, 29.69]	< 0.001
Arterial hypertension = Yes	768 (54.9)	2265 (66.3)	810 (72.5)	< 0.001
Smoking				< 0.001
Current	234 (16.2)	607 (17.1)	293 (25.1)	
Former	625 (43.2)	1581 (44.4)	550 (47)	
Never	588 (40.6)	1369 (38.5)	326 (27.9)	
Diabetes mellitus = Yes	85 (6.2)	242 (7.4)	122 (11.3)	< 0.001
Alcohol intake (g/day)	9.33 [2.69, 21.52]	9.38 [2.7, 22.94]	10.44 [2.78, 26.41]	0.14
*Blood values*				
Non-HDL-cholesterol (mg/dL)	140 [115, 169]	143 [117, 171]	144 [114, 171]	0.19
hsCRP (mg/dL)	0.1 [0.06, 0.23]	0.11 [0.06, 0.25]	0.13 [0.07, 0.3]	< 0.001
*Dental parameters*				
DMFT-Index	17 [14, 21]	19 [16, 23]	21 [17, 24.25]	< 0.001
BOP (%)	2.08 [0, 7.14]	8.33 [2.17, 9.23]	21.05 [9.26, 41.67]	< 0.001
Plaque index (%)	0 [0, 10.71]	8.81 [0, 27.78]	21.74 [5.77, 54.76]	< 0.001

6209 Participants with dental examination. Provided are number and percentage or median and interquartile range. Statistically significant: *p* < 0.05

*BMI* body mass index, *HDL* high-density lipoprotein, *hsCRP* high sensitivity C-reactive protein, *DMFT* decayed, missing and filled teeth, *BOP* bleeding on probing

**Fig. 1:**
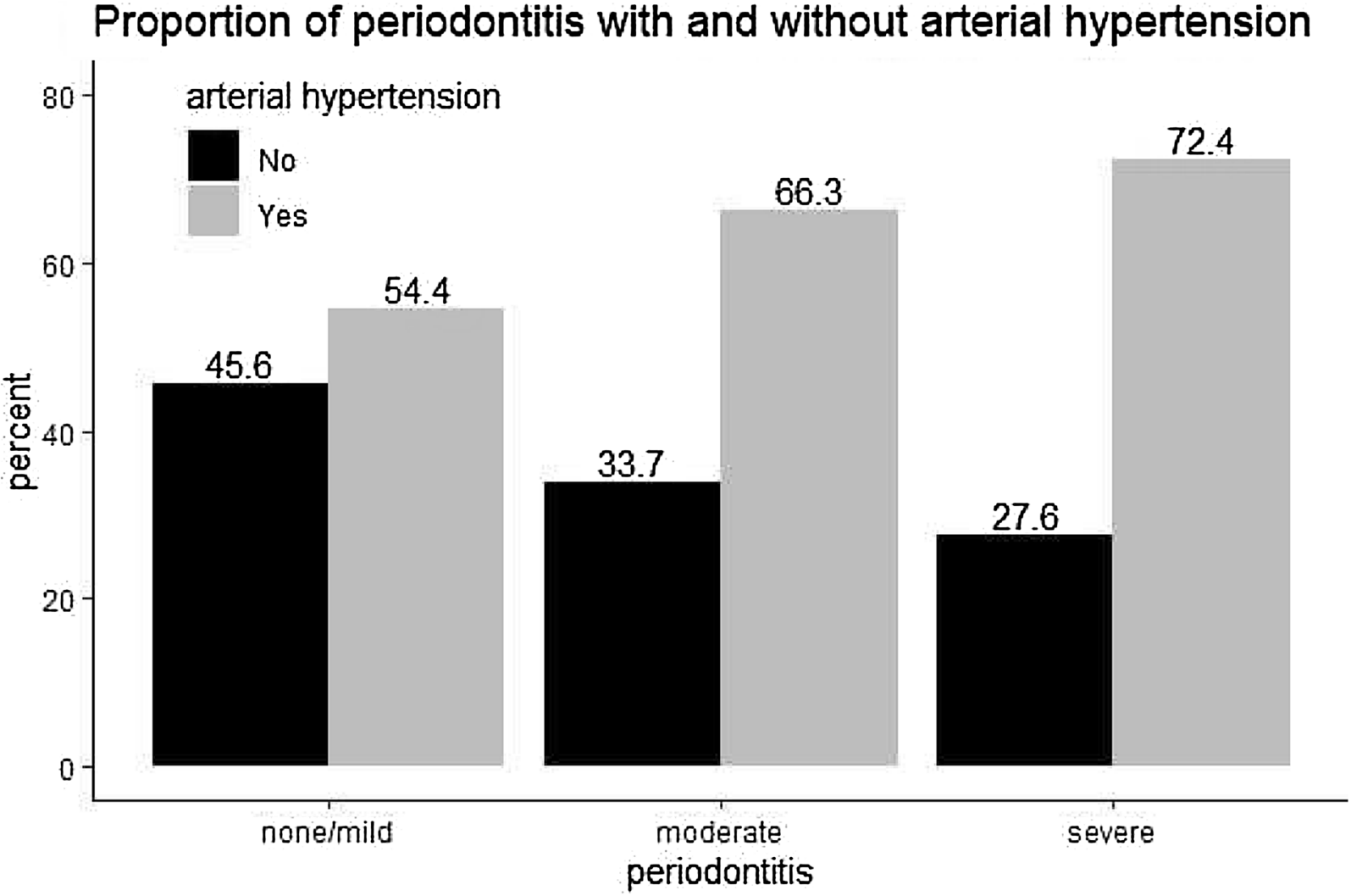
Prevalence of arterial hypertension according to severity grades of periodontitis. Shown are percentages of participants with hypertension (*y*-axis) stratified according to periodontitis severity grades (*x*-axis)

The BOP and the plaque index showed a steep increase as a function of PD severity grades (BOP: From 2.08 to 21.05; PI: From 0 to 21.74), whereas the DMFT index increased more moderately from 17 to 21. Significant differences were detected for the educational level and proportion of women/men (*p* < 0.001). Men (*p* < 0.001) and participants of older age (*p* < 0.001) were more likely to present with severe PD. This pattern was also detected for the BMI (*p* < 0.001). The level of education differed significantly according to PD severity grades (*p* = 0.001, Table 1). Except median non-HDL-cholesterol and alcohol intake, all variables shown in Table 1 differed significantly according to PD severity grades. As is evident from Fig. 1, hypertension was highly prevalent in participants with PD. Almost ¾ of the participants with severe PD and ^2^/_3_ of those with moderate PD had hypertension. As shown in Table 2, the untreated group with normal blood pressure showed an inverse relationship between prevalence and PD severity. In contrast, the prevalence increased considerably from no/mild, moderate to severe periodontitis in the untreated group with hypertension, whereas the group with controlled hypertension showed a much more leveled increase. As is also evident from Table 2, undertreatment of hypertension (untreated and poorly controlled hypertension) was quite common in all studied groups. However, the fraction of participants that was undertreated for hypertension was much larger in participants with severe PD (50.1%) compared with those with no/mild PD (37.4%).

**Table 2:** Effects of antihypertensive medication in groups stratified according to periodontitis severity grades

Periodontitis	Untreated normal blood pressure, *N* (%)	Controlled hypertension, *N* (%)	Untreated hypertension, *N* (%)	Poorly controlled hypertension, *N* (%)	Sum
None/mild periodontitis	696 (50.8)	161 (11.8%)	318 (23.2)	194 (14.2%)	1369
Moderate periodontitis	1324 (40.3%)	463 (14.1%)	898 (27.3%)	602 (18.3%)	3287
Severe periodontitis	356 (33.0%)	182 (16.9%)	327 (30.3%)	214 (19.8%)	1079
All	2376 (41.4%)	806 (14.1%)	1543 (26.9%)	1010 (17.6%)	5735

#### Multiple logistic regression analyses

The odds of arterial hypertension increased significantly along with PD severity in the unadjusted regression model (Table 3, model 1, odds ratio (OR) for moderate PD: 1.65; 95% confidence interval (CI) 1.45–1.87; *p* < 0.001); OR for severe PD: 2.19; 95% CI 1.85–2.59; *p* < 0.001)]. PD was also associated with arterial hypertension in the adjusted models (Table 3—models 2 to 4 and Additional file 3, models 5 and 6). After adjusting for age and sex, participants with moderate and severe PD had significantly higher odds of arterial hypertension compared with periodontal healthy participants (OR for moderate PD: 1.29; 95% CI 1.13–1.48; *p* < 0.001); OR for severe PD: 1.37; 95% CI 1.14–1.64; *p* < 0.001) (Table 3, model 2). Further adjustment for hs-CRP (Table 3, model 3) did hardly change the association, whereas adjusting for age, sex, BMI, diabetes, smoking, education, and alcohol intake (Table 3, model 4) mitigated the association slightly (ORs for moderate/severe PD: 1.30/1.28) but it still remained significant (*p* = 0.001/0.02).

**Table 3:** Association between arterial hypertension and periodontitis severity grades

Parameter	OR per SD (95% CI)	*p* value
*Model 1 (unadjusted)*		
Periodontitis		
None/mild	Reference	
Moderate	1.64 (1.45, 1.87)	< 0.001
Severe	2.19 (1.85, 2.59)	< 0.001
*Model 2 (age, sex)*		
Periodontitis		
None/mild	Reference	
Moderate	1.29 (1.13, 1.48)	< 0.001
Severe	1.37 (1.14, 1.64)	< 0.001
Age	1.98 (1.87, 2.11)	< 0.001
Sex		
Male	Reference	
Female	0.57 (0.51, 0.64)	< 0.001
*Model 3 (age, sex, hsCRP)*		
Periodontitis		
None/mild	Reference	
Moderate	1.29 (1.12, 1.48)	< 0.001
Severe	1.37 (1.14, 1.64)	< 0.001
Age	1.96 (1.84, 2.09)	< 0.001
Sex		
Male	Reference	
Female	0.58 (0.51, 0.65)	< 0.001
hsCRP	1.33 (1.2, 1.47)	< 0.001
*Model 4 (age, sex, BMI, diabetes, smoking, education, alcohol intake)*		
Periodontitis		
None/mild	Reference	
Moderate	1.3 (1.11, 1.52)	0.001
Severe	1.28 (1.04, 1.59)	0.02
Age	1.99 (1.85, 2.14)	< 0.001
Sex		
Male	Reference	
Female	0.62 (0.54, 0.72)	< 0.001
BMI	1.79 (1.65, 1.94)	< 0.001
Diabetes		
No	Reference	
Yes	2.18 (1.55, 3.08)	< 0.001
Smoking		
Never	Reference	
Current	0.81 (0.67, 0.97)	0.03
Former	0.92 (0.79, 1.07)	0.28
Education		
Low	Reference	
Medium	0.89 (0.6, 1.3)	0.53
High	0.72 (0.49, 1.06)	0.1
Alcohol intake (g/day)	0.99 (0.92, 1.06)	0.73

5735 participants with complete data. Shown are odds ratio (OR) per SD and 95% confidence interval (CI) resulting from crude and progressively adjusted logistic regression models as described in the Methods section. Statistically significant: *p* < 0.05

*BMI*,  body mass index, *hsCRP*, high sensitivity C-reactive protein

Further models including adjustments for age, sex, BMI, diabetes, smoking, and education (Additional file 3, model 5) or age, sex, BMI, diabetes, smoking, education, and hsCRP (Additional file 3, model 6) provided similar results as model 4 (Table 3), which included adjustments for age, sex, BMI, diabetes, smoking, education, and alcohol intake.

## Discussion

The current study shows an association between PD and arterial hypertension that was independent of age, sex, diabetes, BMI, smoking, educational level, and alcohol intake. Both, moderate and severe PD were associated with arterial hypertension and people with PD exhibited higher median SBP when compared with none/mild PD. Several previous cross-sectional studies and meta-analyses support a positive association between periodontal disease and arterial hypertension [20–24]. The probably largest came from the National Health and Nutrition Examination Survey (NHANES) and demonstrated that people with PD on average had higher blood pressure and more often arterial hypertension than orally healthy people [20]. Significant associations with SBP and arterial hypertension were found in all periodontal measurements. After adjustment for a range of covariates, individuals with 10% increased gingival bleeding had 1.1 times higher odds of arterial hypertension. Furthermore, participants with PD had a higher probability of arterial hypertension in both sexes, but a clear linear trend was observed only in men [20].

A unique observation of our study is that PD severity was strongly linked to different forms of arterial hypertension depending on antihypertensive treatment efficiency. As might be expected, optimal periodontal health was linked to the untreated group with normal blood pressure that showed an inverse relationship between prevalence and PD severity. The worst periodontal health was linked to the untreated hypertensive group, whereas the groups with controlled and uncontrolled hypertension showed intermediate results. We observed that undertreatment of hypertension (untreated and poorly controlled hypertension) was quite common in all groups. However, the fraction of participants that was undertreated for hypertension was particularly large in the group with severe PD.

Our results show that PD is one of the morbidities showing a strong association between inflammation and blood pressure. Czesnikiewicz-Guzik et al. showed that Th1 immune responses induced by bacterial antigens derived from *Porphyromonas gingivalis* increased the sensitivity to subpressor pro-hypertensive insult in mice and provided a mechanistic link between PD-associated chronic infection and hypertension [34]. Moreover, it could be demonstrated that intensive periodontal treatment improved the periodontal status and lowered blood pressure in humans [35].

A causal relationship between PD and arterial hypertension cannot not be concluded from our study due to the cross-sectional design. However, a recent Mendelian randomization study demonstrated that periodontitis-linked SNPs were associated with increased BP, suggesting that the relationship between PD and arterial hypertension may in fact be causal [35]. In addition, the oral microbiome’s ability to reduce inorganic nitrate to nitrite and nitric oxide may provide a plausible mechanism linking PD to arterial hypertension [19].

Our study has a number of strengths: (1) it is one of the largest studies on this topic to date. (2) The diagnosis of arterial hypertension was based on a detailed medical history, with focus on antihypertensive drugs, two blood pressure measurements and self-reports. (3) Due to the mono-centric study design, blood pressure measurement could be performed with the same device by the same calibrated and certified study nurses. (4) The measurements were performed routinely on the right arm after 5 min rest while sitting. (5) The participants were randomly recruited from the residents’ registry office. (6) an unusually large number of potential confounders were assessed, and (7) PD was diagnosed based on a full-mouth examination according to internationally accepted guidelines, whereas NHANES and other large surveys mostly employed half- or partial-mouth protocols that may have tended to underestimate the prevalence of PD [36].

On the other hand, also limitations have to be noted. The distributions of baseline characteristics of the study participants shown in Table 1 and the distribution of missing data shown in (Additional files 1 and 2) suggest no bias with educational level as one obvious exception. Participants with low educational level were underrepresented, while participants with high educational level were overrepresented, which may potentially be explained by the exclusion of people with “poor German language skills” (not being able to comprehend the consent forms) and a participation bias favoring participation of highly over lowly educated people. It is well-known that a low educational level may have negative effects on the general health status in a similar manner as low SES [37]. This would lead to the expectation of a healthier study population compared to the general population. However, a higher participation rate of lowly educated people would rather have strengthened than weakened the association. The values observed for prevalence of exposure and outcome were similar to the expected values from nationally representative surveys (severe PD 19.8 vs. 21.5% in this study and AH 67.1 vs. 64.8% in this study) [38, 39]. Thus, we are confident that our findings have a high degree of external validity and generalisability. On the other hand, we could not implement the most recent PD case classification [30, 40], because our study started in 2016 before the new classification was issued. Therefore, our examination routine was optimized to stage periodontitis according to the 2012 classification [31]. However, the 2012 classification has been used in virtually all previous epidemiological studies related to the topic of this work. Thus, its use in our present study made comparisons with NHANES and other previously published studies straightforward. In addition, the BOP and PI indices used in this study were simplified, which was necessary due to the restricted time, which had been allocated to the dental examination in the HCHS.

In conclusion, this large cross-sectional study demonstrates an association between PD and arterial hypertension that is independent of age, sex, diabetes, smoking, BMI, educational level, and alcohol intake. Moreover, undertreatment of hypertension was more common in people with severe PD compared with periodontally more healthy people. Since arterial hypertension is a major preventable risk factor of cardiovascular disease and PD has been linked to increased risk of heart disease and stroke, these results are of clinical relevance.

## Supplementary Information

**Additional file 1.** Sampling flow chart.

**Additional file 2.** Missing data for key variables.

**Additional file 3.** Additional multivariable regression models.

## Data Availability

The data sets generated and/or analyzed during the current study are not publicly available due privacy/ethical restrictions but are available from the corresponding author on reasonable request.

## References

[1] Flack JM, Adekola B (2020). Blood pressure and the new ACC/AHA hypertension guidelines. Trends Cardiovasc Med.

[2] European Society of Cardiology (2018). ESC/ESH Guidelines for the management of arterial hypertension. Eur Heart J.

[3] Zhou B, Carrillo-Larco RM, Danaei G, Riley LM, Paciorek CJ, Stevens GA, Gregg EW, Bennett JE, Solomon B, Singleton RK, Sophiea MK (2021). Worldwide trends in hypertension prevalence and progress in treatment and control from 1990 to 2019: a pooled analysis of 1201 population-representative studies with 104 million participants. Lancet.

[4] Buttar HS, Li T, Ravi N (2005). Prevention of cardiovascular diseases: role of exercise, dietary interventions, obesity and smoking cessation. Exp Clin Cardiol.

[5] Jiang SZ, Lu W, Zong XF, Ruan HY, Liu Y (2016). Obesity and hypertension. Exp Ther Med.

[6] Guyton AC (1991). Blood pressure control—special role of the kidneys and body fluids. Science.

[7] Beevers G, Lip GY, O'Brien E (2001). The pathophysiology of hypertension. BMJ.

[8] Drummond GR, Vinh A, Guzik TJ, Sobey CG (2019). Immune mechanisms of hypertension. Nat Rev Immunol.

[9] Guzik TJ, Hoch NE, Brown KA, McCann LA, Rahman A, Dikalov S, Goronzy J, Weyand C, Harrison DG (2007). Role of the T cell in the genesis of angiotensin II-induced hypertension and vascular dysfunction. J Exp Med.

[10] Norlander AE, Madhur MS, Harrison DG (2018). The immunology of hypertension. J Exp Med.

[11] Wenzel U, Turner JE, Krebs C, Kurts C, Harrison DG, Ehmke H (2016). Immune mechanisms in arterial hypertension. J Am Soc Nephrol.

[12] Wenzel UO, Ehmke H, Bode M (2021). Immune mechanisms in arterial hypertension. Rec Adv Cell Tissue Res.

[13] Darnaud C, Thomas F, Pannier B, Danchin N, Bouchard P (2015). Oral health and blood pressure: the IPC cohort. Am J Hypertens.

[14] Chen MX, Zhong YJ, Dong QQ, Wong HM, Wen YF (2021). Global, regional, and national burden of severe periodontitis, 1990–2019: an analysis of the Global Burden of Disease Study 2019. J Clin Periodontol.

[15] Könönen E, Gursoy M, Gursoy UK (2019). Periodontitis: a multifaceted disease of tooth-supporting tissues. J Clin Med.

[16] Zambon JJ (1996). Periodontal diseases: microbial factors. Ann Periodontol.

[17] Silva N, Abusleme L, Bravo D, Dutzan N, Garcia-Sesnich J, Vernal R, Hernandez M, Gamonal J (2015). Host response mechanisms in periodontal diseases. J Appl Oral Sci.

[18] Cekici A, Kantarci A, Hasturk H, Van Dyke TE (2014). Inflammatory and immune pathways in the pathogenesis of periodontal disease. Periodontol.

[19] Bryan NS, Tribble G, Angelov N (2017). Oral microbiome and nitric oxide: the missing link in the management of blood pressure. Curr Hypertens Rep.

[20] Tsakos G, Sabbah W, Hingorani AD, Netuveli G, Donos N, Watt RG, D'Aiuto F (2010). Is periodontal inflammation associated with raised blood pressure? Evidence from a National US survey. J Hypertens.

[21] Tsioufis C, Kasiakogias A, Thomopoulos C, Stefanadis C (2011). Periodontitis and blood pressure: the concept of dental hypertension. Atherosclerosis.

[22] Zeigler CC, Wondimu B, Marcus C, Modéer T (2015). Pathological periodontal pockets are associated with raised diastolic blood pressure in obese adolescents. BMC Oral Health.

[23] Martin-Cabezas R, Seelam N, Petit C, Agossa K, Gaertner S, Tenenbaum H, Davideau JL, Huck O (2016). Association between periodontitis and arterial hypertension: a systematic review and meta-analysis. Am Heart J.

[24] Munoz Aguilera E, Suvan J, Buti J, Czesnikiewicz-Guzik M, Barbosa Ribeiro A, Orlandi M, Guzik TJ, Hingorani AD, Nart J, D’Aiuto F (2020). Periodontitis is associated with hypertension: a systematic review and meta-analysis. Cardiovasc Res.

[25] Jagodzinski A, Johansen C, Koch-Gromus U, Aarabi G, Adam G, Anders S, Augustin M, der Kellen RB, Beikler T, Behrendt CA, Betz CS (2020). Rationale and design of the Hamburg city health study. Eur J Epidemiol.

[26] von Elm E (2008). The Strengthening the Reporting of Observational Studies in Epidemiology (STROBE) statement: guidelines for reporting observational studies. J Clin Epidemiol.

[27] Lamprecht R, Rimmele DL, Schnabel RB, Heydecke G, Seedorf U, Walther C, Mayer C, Struppek J, Borof K, Behrendt CA, Cheng B (2022). Cross-sectional analysis of the association of periodontitis with carotid intima media thickness and atherosclerotic plaque in the Hamburg City health study. J Periodontal Res.

[28] Holtfreter B, Samietz S, Hertrampf K, Aarabi G, Hagenfeld D, Kim TS, Kocher T, Koos B, Schmitter M, Ahrens W, Alwers E (2020). Design and quality control of the oral health status examination in the German National Cohort (GNC). Bundesgesundheitsblatt Gesundheitsforschung Gesundheitsschutz.

[29] Silness J, Löe H. Periodontal disease in pregnancy II. Correlation between oral hygiene and periodontal condition. Acta Odontol Scand. 1964;22(1):121–35. 10.3109/00016356408993968 14158464

[30] Chapple IL, Mealey BL, Van Dyke TE, Bartold PM, Dommisch H, Eickholz P, Geisinger ML, Genco RJ, Glogauer M, Goldstein M, Griffin TJ (2018). Periodontal health and gingival diseases and conditions on an intact and a reduced periodontium: consensus report of workgroup 1 of the 2017 World Workshop on the Classification of Periodontal and Peri-Implant Diseases and Conditions. J Periodontol.

[31] Eke PI, Page RC, Wei L, Thornton-Evans G, Genco RJ (2012). Update of the case definitions for population-based surveillance of periodontitis. J Periodontol.

[32] Heatherton TF, Kozlowski LT, Frecker RC, Fagerstrom KO (1991). The Fagerström test for nicotine dependence: a revision of the Fagerstrom Tolerance Questionnaire. Br J Addict.

[33] Czesnikiewicz-Guzik M, Nosalski R, Mikolajczyk TP, Vidler F, Dohnal T, Dembowska E, Graham D, Harrison DG, Guzik TJ (2019). Th1-type immune responses to *Porphyromonas gingivalis* antigens exacerbate angiotensin II-dependent hypertension and vascular dysfunction. Br J Pharmacol.

[34] Czesnikiewicz-Guzik M, Osmenda G, Siedlinski M, Nosalski R, Pelka P, Nowakowski D, Wilk G, Mikolajczyk TP, Schramm-Luc A, Furtak A, Matusik P (2019). Causal association between periodontitis and hypertension: evidence from Mendelian randomization and a randomized controlled trial of non-surgical periodontal therapy. Eur Heart J.

[35] Albandar JM (2011). Underestimation of periodontitis in NHANES surveys. J Periodontol.

[36] Ross CE, Wu CL. Education, age, and the cumulative advantage in health. J Health Soc Behav. 1996:104–20. 8820314

[37] Diederichs C, Neuhauser H (2017). The incidence of hypertension and its risk factors in the German adult population: results from the German National Health Interview and Examination Survey 1998 and the German Health Interview and Examination Survey for Adults 2008–2011. J Hypertens.

[38] Jordan, A. R., Micheelis, W. Fünfte Deutsche Mundgesundheitsstudie (DMS V). Deutscher Ärzteverlag, 2016; p. 617.

[39] Caton JG, Armitage G, Berglundh T, Chapple IL, Jepsen S, Kornman KS, Mealey BL, Papapanou PN, Sanz M, Tonetti MS (2018). A new classification scheme for periodontal and peri-implant diseases and conditions—introduction and key changes from the 1999 classification. J Periodontol.

[40] Tonetti MS, Greenwell H, Kornman KS (2018). Staging and grading of periodontitis: framework and proposal of a new classification and case definition. J Periodontol.

