# Supplementary material for: Cross-sectional study on the association of periodontitis with arterial hypertension in the Hamburg City Health Study
Source: Eur J Med Res. 2022 Sep 16;27:181. doi: 10.1186/s40001-022-00811-y (PMC9479239; doi:10.1186/s40001-022-00811-y)
Supplement: Supplementary file 1 — Additional file 1. Sampling flow chart. [file 40001_2022_811_MOESM1_ESM.docx]

**Sampling flow chart**

The ongoing Hamburg City Health Study (HCHS) is projected to include 45,000 randomly drawn residents of Hamburg, Germany, who will be followed up for at least 6 years. Periodontitis severity grades were available for a random sample of 6,209 out of 10,000 participants recruited between February 2016 and November 2018. Blood pressure values were available for 5,934 participants, of which 3,843 participants (64.8%) had hypertension.
