# Supplementary material for: Cross-sectional study on the association of periodontitis with arterial hypertension in the Hamburg City Health Study
Source: Eur J Med Res. 2022 Sep 16;27:181. doi: 10.1186/s40001-022-00811-y (PMC9479239; doi:10.1186/s40001-022-00811-y)
Supplement: Supplementary file 2 — Additional file 2. Missing data for key variables. [file 40001_2022_811_MOESM2_ESM.docx]

**Missing data for key variables**

| **Variable** | **Missing N (%) All^1^** | **Missing N (%) Periodontitis^2^** |
| --- | --- | --- |
| Sex | 0 (0) | 0 (0) |
| Age | 0 (0) | 0 (0) |
| Education | 561 (5.6) | 285 (4.6) |
| Hypertension | 467 (4.7) | 275 (4.4) |
| Smoking | 54 (0.54) | 36 (0.58) |
| Periodontitis | 3,791 (37.9) | N.a. |

^1^N=10,000; ^2^N=6,209
