# Supplementary material for: Cross-sectional study on the association of periodontitis with arterial hypertension in the Hamburg City Health Study
Source: Eur J Med Res. 2022 Sep 16;27:181. doi: 10.1186/s40001-022-00811-y (PMC9479239; doi:10.1186/s40001-022-00811-y)
Supplement: Supplementary file 3 — Additional file 3. Additional multivariable regression models. [file 40001_2022_811_MOESM3_ESM.docx]

**Additional multivariable regression models**

| **Parameter** | | **OR per SD (95% CI)** | **p-value** |
| --- | --- | --- | --- |
| **Model 5 (adjusted for age, sex, BMI, diabetes, smoking, education)** | | | |
| Periodontitis | |  |  |
| None/mild | Reference |  |  |
| Moderate | 1.25 (1.07, 1.45) | 0.004 |  |
| Severe | 1.30 (1.06, 1.60) | 0.01 |  |
| Age | | 1.99 (1.85, 2.13) | < 0.001 |
| Sex | |  |  |
| Male | Reference |  |  |
| Female | 0.61 (0.53, 0.67) | < 0.001 |  |
| BMI | | 1.76 (1.63, 1.90) | < 0.001 |
| Diabetes | |  |  |
| No | Reference |  |  |
| Yes | 2.35 (1.68, 3.29) | < 0.001 |  |
| Smoking | |  |  |
| Never | Reference |  |  |
| Current | 0.84 (0.7, 1.01) | 0.06 |  |
| Former | 0.95 (0.82, 1.09) | 0.47 |  |
| Education | |  |  |
| Low | Reference |  |  |
| Medium | 0.97 (0.69, 1.37) | 0.86 |  |
| High | 0.77 (0.55, 1.09) | 0.15 |  |
| **Model 6 (adjusted for age, sex, BMI, diabetes, smoking, education, hsCRP)** | | | |
| Periodontitis | |  |  |
| None/mild | Reference |  |  |
| Moderate | 1.25 (1.08, 1.46) | < 0.005 |  |
| Severe | 1.31 (1.07, 1.61) | 0.01 |  |
| Age | | 1.99 (1.85, 2.13) | < 0.001 |
| Sex | |  |  |
| Male | Reference |  |  |
| Female | 0.61 (0.53, 0.7) | < 0.001 |  |
| BMI | | 1.75 (1.61, 1.89) | < 0.001 |
| Diabetes | |  |  |
| No | Reference |  |  |
| Yes | 2.29 (1.63, 3.22) | < 0.001 |  |
| Smoking | |  |  |
| Never | Reference |  |  |
| Current | 0.84 (0.7, 1.01) | 0.06 |  |
| Former | 0.93 (0.81, 1.08) | 0.36 |  |
| Education | |  |  |
| Low | Reference |  |  |
| Medium | 0.99 (0.7, 1.39) | 0.94 |  |
| High | 0.79 (0.56, 1.12) | 0.19 |  |
| hsCRP | | 1.06 (0.98, 1.14) | 0.16 |

5,735 participants with complete data. Shown are odds ratio (OR) per SD and 95% confidence interval (CI) resulting from crude and progressively adjusted logistic regression models as described in the Methods section. Statistically significant: p < 0.05.

BMI = body mass index, hsCRP = high sensitivity C-reactive protein
